# ‘We are unlikely to return to the same world, and I do not want it to destroy my future.’ Young people's worries through the outbreak of the COVID‐19 pandemic

**DOI:** 10.1111/cfs.12878

**Published:** 2021-10-10

**Authors:** Ragnhild Bjørknes, Gro Mjeldheim Sandal, Silje Mæland, Ellen Haug, Stine Lehmann

**Affiliations:** ^1^ Department of Health Promotion and Development, Faculty of Psychology The University of Bergen Bergen Norway; ^2^ Department of Psychosocial Science, Faculty of Psychology The University of Bergen Bergen Norway; ^3^ Department of Global Public Health and Primary Care, Faculty of Medicine The University of Bergen Bergen Norway; ^4^ Research Unit for General Practice in Bergen The Norwegian Research Center, NORCE Bergen Norway; ^5^ Department of Education NLA University College Bergen Norway

**Keywords:** COVID‐19, lost youth, mental health, outlook in life, worries, youth

## Abstract

This study aims to explore what worries youth were having during the seventh to ninth week of the COVID‐19 lockdown. Our findings build on the responses to an open‐ended survey question from 1314 youths. The worries covered three main themes: ‘That my mom dies, then I am left all alone’: worries related to COVID‐19 virus infection; ‘To me, this is lost youth’: worries about the consequences of measures for the present life and near future; and ‘I will face a very difficult life in the future’: worries about the consequences of measures for the outlook on life. Young people worried that the measures would have a huge impact on their present life and outlook on life. The costs of restriction measures were unevenly distributed and indicated that the measures affected their mental health. Listening to youth voices during the pandemic is important for practitioners, educators and policymakers. The results indicate that the threshold for closing schools also including the provision of distance learning should be kept high. Social and health services for youth should offer early intervention and be prepared for an escalation in mental health problems in the imminent future.

## 1 INTRODUCTION

It is expected that the global crisis caused by the COVID‐19 pandemic, including the disease and prolonged social distancing, will have major impacts on young people's wellbeing and future mental health (European Centre for Disease Prevention and Control [ECDC], 2020). This study explores what worries the future among youth aged 12 to 19 years after 7–9 weeks (April and May) of COVID‐19 lockdown in Bergen, Norway.

During the outbreak of the COVID‐19 pandemic in the spring of 2020, a national suppression strategy with different measures to suppress the spread of infection was implemented (Norwegian Government, 2021). As in most other countries, schools in Norway replaced physical teaching with digital homeschooling. Children were assigned to homeschooling, and about half of the parents were working from home. Also, most of the organized leisure activities for youth closed as part of the measures. On the societal level, strict rules on social distancing were introduced, including avoiding physical meetings with people outside the nearest family. Overall, the measures to repress the spreading of the virus led to major interferences in everyday life of youth with a dramatic decrease in social contact and time spent with friends, teachers and in leisure activities (for a detailed description of the main measures implemented during these weeks, see Christensen & Lægreid, 2020).

The physical distancing measures that removed many sources of social connection from youth's lives are of concern because adolescence is in a sensitive period for identity formation characterized by an enhanced need for peer interaction. Friends facilitate young people's transition to independent adulthood by strengthening their sense of social self‐identity and relationships with their peer group (Blakemore & Mills, 2014; Brown et al., 1986). Studies from several European countries have reported that children and adolescents missed their friends, and they longed for social life at school and in leisure activities during the pandemic (Branquinho et al., 2020; Buzzi et al., 2020; Commodari & La Rosa, 2020; Nøkleby et al., 2021). In addition, other Norwegian and international studies have identified an increase in children's depressive symptoms, anxiety, irritability, boredom and stress during the lockdown (ECDC, 2020; Loades et al., 2020; Nøkleby et al., 2021). This adds to an increasing concern that the lockdown of society, particularly schools, could have a prolonged adverse effect on the educational, physical and mental health of youths (Clemens et al., 2020; Commodari & La Rosa, 2020; ECDC, 2020; Guessoum et al., 2020; Orben et al., 2020).

Studies examining how the dramatic and sudden shift from everyday routines to days regulated by measures has affected youth are emerging. In a Norwegian study of 244 youth aged 13–20 years old, Dyregrov et al. (2020) found that youth's risk perceptions and experiences in the COVID‐19 period were mostly related to their concern about infecting someone close to them. They were also concerned about their future, particularly education and social life. These findings align with a qualitative study from the United Kingdom where youth worries centred on the possibility of infecting vulnerable others, their education and the future (Larcher et al., 2020). Dyregrov and colleagues further found that only a few were very worried about getting the virus themself and that significantly more girls than boys were overall concerned about COVID‐19. The latter findings are in line with the survey results from the COVID‐19 Young study (Lehmann et al., 2021), where youth aged 12 to 19 years old participated. Of these, a little over half (54%) reported being somewhat or very worried that the pandemic would lead to a more difficult future for themselves. Seven percent were worried about getting infected, whereas 53% were worried about spreading the virus to family members. In addition, a substantial proportion (54%) of the participants reported negative consequences of schools getting closed, and 63% reported learning less during homeschooling. Girls, older youth, migrant youth and youth with lower socio‐economic status (SES) experienced the lockdown as the most challenging (Lehmann et al., 2021). For young people, the impact of COVID‐19 suppressive measures on their mental health could be more detrimental in the long run than the infection itself (Shukla et al., 2021). Understanding early symptoms of mental health challenges such as worries, and the content and salience of different worries, might inform health interventions that could target vulnerable youth. Young people contribute with important experiences related to their worries (Branquinho et al., 2020; Dyregrov et al., 2020), and the voice of young people should be heard and valued (Nations Convention on the Rights of the Child, 1989), especially during a pandemic.

In this study, the following research question was raised: What worries about the future did youth aged 12 to 19 years have during the seventh to ninth week of the COVID‐19 lockdown? The current study examines in‐depth the responses to an open‐ended question about worries among the participants in the COVID‐19 Young study. We believe that the qualitative data add important value to findings from the quantitative data already published (Lehmann et al., 2021). The advantage of open‐ended questions in epidemiological surveys, rather than restricting their use to qualitative interviews, is the breadth and representativeness of coverage they provide (Singer & Couper, 2017). Thus, the analysis of the respondents' written accounts may provide important insights and allow for the voices of a large number of youths to be heard.

## 2 METHODS

### 2.1 Design

The COVID‐19 Young is a study of youth aged 12–19 years. The study is part of a larger population‐based survey in Bergen/Norway (Bergen in ChangE‐study: Mæland et al., 2021). The study was conducted in April/May 2020 to map health and everyday life during the pandemic. Using data from this study, we have explored how the implemented nonpharmaceutical interventions in Norway have affected adults (*n* = 29,535, 18–99 years) and youth health, sleep, physical activity, health‐care use and family dynamics (*n* = 2997, 12–19 years). The COVID‐19 Young comprises two samples: Cohort 1 consists of youths aged 12–15 years whose parents had participated in the Bergen in ChangE‐study and consented that their child could participate. These parents were more often females, older, had higher educational attainment and household income, and had less often shared residence for the child when compared with non‐consenting parents (Lehmann et al., 2021). Upon consent, parents provided contact information for the youth, and a total of 1565 youth in Cohort 1 were contacted. Cohort 2 consists of youths aged 16–19 years, attending high schools in Bergen municipality. The county provided phone numbers from their contact registers, and a total of 5947 youth was contacted. The data collection for both cohorts started 27^th^ of April 2020, during the seventh week of lockdown, and closed on 11^th^ of May. The invitation procedures were the same for Cohort 1 and 2. Youth were recruited via SMS with a link to a secure online platform, containing an information letter and a 15‐min online survey. Two SMS reminders were given. Participants were included in a lottery for a new cellphone. Of the 7512 youths that were invited to participate, 843 (54%) in Cohort 1 and 2154 (36%) in Cohort 2 responded, yielding a total of 2997 (40%) youths completing the survey.

### 2.2 Measures

#### 2.2.1 Demographics

Demographic data included gender, age, and country of birth, and were collected from the youth self‐reports when completing the online survey.

#### 2.2.2 Worries about the future

Firstly, the participants were asked to indicate their degree of agreement with the following statement: ‘I'm worried that the outbreak will lead to a more difficult future for me’ Responses were given on a 4‐point scale. Participants who replied ‘Yes, somewhat’ or ‘Yes very much’ were given a follow‐up, open‐ended question: ‘You've replied that you are worried that the outbreak will lead to a more difficult future for you. What are you worried about?’

### 2.3 Participants

Of the youth participating in the COVID‐19 Young, 1531 (54%) reported that they were somewhat or very worried that the outbreak would lead to a more difficult future for themselves. A total of 1314 informants answered the open‐ended question. Of these respondents, 23.5% (*n* = 306) attended middle school, and 76.5% (*n* = 1003) were high school students. The sample comprised 37.5% boys (*n* = 492) and 61.5% girls (*n* = 808), and 7.7% (*n* = 101) were ethnic minority youth.

### 2.4 Ethics

Youth consented to participate by ticking the consent form at the start of the survey. The Regional Committee for Medical and Health Research Ethics, Western Norway approved the study (project number 131560).

### 2.5 Analyses

The written accounts had between 10 and 1000 characters, with an average of 250 characters. There were no restrictions on the length of the accounts for the youths. We used systematic text condensation (STC) (Malterud, 2012), in the analyses of the young people's written accounts. STC is a descriptive approach, aiming to present the participants' experiences as expressed by them, rather than searching for potential underlying meanings in what is said (Malterud, 2012). Before opening the data set, all analysts wrote down subjective preconceptions about youth future worries. The method involves four stages for thematic cross‐case analysis (Malterud, 2012), and throughout this process, the analysts were conscious of their written subjective preconceptions. In the first stage, two analysts (RB and SM) individually read the written accounts to obtain an overall impression and look for preliminary themes. Each analyst identified eight preliminary themes (four to eight recommended in STC, Malterud, 2012). Each preliminary theme was discussed to find possible agreement or disagreement between the analysts and to understand the content and meaning of the theme. Eight themes were agreed upon to prioritize in the next step. In stages two and three, the first author coded all 1314 meaning units according to the themes. Next, all analysts (RB, SM, SL and GMS) collaborated in an iterative process in discussing the themes and coding of meaning units. Some meaning units were recoded, and themes were negotiated and refined accordingly. In this way, the preliminary themes were processed and formed into three code groups and five subcategories informed by the meaning units (Malterud, 2012). The analysts then condensed the meaning unit within each code group in a first‐person ‘one‐voice’ quotation. By continuously checking with preconceptions, we made sure to not mirror preconceptions. In the fourth stage, the first‐person text was re‐written into an analytical third‐person conceptual text, representing youths' worries for the future. This was done to recontextualize the experiences. The conceptual descriptions and their headings, and a further concentration of the code group are displayed in Section 3.

## 3 RESULTS

The youth reported several types of worries during the seventh to ninth week of the COVID‐19 lockdown, and the results are summed up in Table 1 below.

**TABLE 1:** Overview of themes and subthemes

Themes	Subthemes
‘That my mom dies, then I am left all alone’. Worries related to the COVID‐19 virus infection.	Worries about getting infected by COVID‐19 themselves or close ones.
‘To me, this is lost youth’. Worries about the consequences of measures for the present life and near future.	Worries about friendship, contact with family, and everyday life.
	Worries about mental health.
	Worries about learning outcomes.
‘I will face a very difficult life in the future’. Worries about the consequences of measures for the outlook on life.	Worries about future education.
	Worries about economic safety in the future.

Many of the answers from the youth included themes that ran across the three main topics. This shows that perspectives and worries are related across the main topics and many of the concerns followed as a subsequent chain of worries. The respective contributors have been assigned pseudonyms.

### 3.1 ‘That my mom dies, then I am left all alone’: Worries related to the COVID‐19 virus infection

Of the total sample, very few youths worried about issues related to the covid‐19 virus infection. The comments from these responders were coded into one theme.

#### 3.1.1 Worries about getting infected by COVID‐19 themselves or close ones

Of the young participants in our study, remarkably few were worried about the possibility of getting infected by COVID‐19 themselves.

Those few who worried about becoming ill themselves or even die mainly had underlying diseases (e.g., respiratory illnesses). A larger, but still somewhat small proportion of youth described that they were worried that their family members would get infected by COVID‐19. Some described that they were worried because family members were in a medical risk group. Fear of losing a parent and other close adults', leaving them alone, and even becoming orphans, were themes in some of the comments.

One youth said: ‘If my grandparents die, my future is going to be harder, mostly because I love them very much and we have a lot of traditions that are important to me’. Another youth explained the worries like this: ‘That people in my family die, and I become all alone’.

Fear of being responsible for infecting loved ones who would then die was also mentioned in comments. This implied that the youth became infected and passed the virus on to close family members. For the youth, this would imply a double burden of losing significant persons and for being to blame for their death. Thus, the youth needed to avoid being infected or keep their distance to family members at risk.

### 3.2 ‘To me, this is lost youth’: Worries about the consequences of measures for the present life and near future

The large majority of the respondents expressed worries about the impact of the measures on their present lives and near future. These responses could be divided into three subthemes. A line that went across these themes was the costs of social distancing, closed schools and organized leisure activities. These costs were related to their loss of daily routines and important events during adolescence.

#### 3.2.1 Worries about friendship, contact with family and everyday life

Many youths worried about the consequences social distancing would have on friendship and family relations. They worried that they would not return to their normal life, where schools were open, and they could meet friends. Subsequently, the measures would have severe consequences for their social life. Some of the youth described loss of specific events during their adolescence, and a fear/worry of not being able to live their adolescent lives fully.

Some also raised the burden of always taking precautions for instance when meeting grandparents. Many young people expressed that their lives were being put ‘on hold’ and daily life seemed distant. This fueled worries that valuable adolescent years potentially could be lost. Losing important events and moments such as social events, birthdays, summer vacations, religious ceremonies and graduation celebrations made them feel trapped at home in isolation. The unpredictability about the duration and impact of repressive measures seemed to stir the worries about long‐term severe consequences. One potentially negative consequence voiced by many was that schools closing for longer periods would lead to loss of social meeting points and impaired friendships with classmates. The prolonged lockdown made many worry about when, if ever, they could socialize freely again, including spending time with many friends at the same time. Several of the youth revealed stories about how social media had become a more important arena for friendship and social interaction during the pandemic, and particularly during lockdown. Importantly, some of the youth wrote that they did not feel comfortable and capable of socializing on such platforms and that this left them even more isolated. One young person put it like this: ‘That the friends I had before the 12th of March are slipping away from me, and that it will be a lonely summer … I worry that my future will be affected by it’.

#### 3.2.2 Worries about mental health

Some of the youth indicated that the measures affected their mental health. Youth with mental health problems prior to the pandemic experienced or were anxious about relapse or escalation of their problems. Among these mental health problems were ADHD, concentration problems, depression and social anxiety. Some of the youth worried that the lockdown would interrupt contact with mental health professionals that had been established before the pandemic. Several mentioned that schools played an important role in their therapeutic regimes. For example, one youth with anxiety emphasized that going to school was important as a practice to become less tense in social situations. Another youth with ADHD stressed that individual follow‐up from teachers was important to manage schoolwork.

Several of the youth reported that family problems had evolved or aggravated during the lockdown, and they had experienced or were worried that such difficulties could negatively impact their mental health. For this group, going to school represent a time‐out from a strenuous family relationship. One commented: ‘After Covid‐19, my life has only become more difficult. I have started to get suicidal thoughts again and have started self‐injuring again. I feel that it is difficult at home, and now I don't have a school where I can hide’.

Even youth who did not appear to have mental health challenges prior to the pandemic worried about the psychological impact of the social distancing measures and in particular, homeschooling. A repeated theme was worrying that mental health problems would interfere with the progress at school and destroy future educational and work opportunities. Also, several youths emphasized that they worried that the pandemic and preventive measures could lead to permanent and irreversible mental health problems that again would seriously impact on their future life.

One mentioned: ‘That I do not learn what I should, and therefore I will struggle for the rest of my life. My psychological health becomes worse and I worry that it will not recover after Corona has ended’.

#### 3.2.3 Worries about learning outcomes

Many youths are worried about their learning outcomes due to school closure. Typically, they worried about learning less from homeschooling and digital teaching. Several youths worried that their grades would go down with distance learning. Others worried that they could not finish their practice module in high school due to the lockdown of working places. Some stated that their motivation had declined and they experienced symptoms of fatigue and difficulties concentrating. A few participants mentioned that they were afraid to drop out of school.

One comment that summarized many of the worries was stated like this: ‘(I am worried about) all the schooling that I miss out on because of homeschooling. On a normal school day, we had around 10 hours of school, now I have almost three hours of school. I feel that I am losing the education I am entitled to.’

### 3.3 ‘I will face a very difficult life in the future’: Worries about the consequence of measures for the outlook on life

A thematic line that went across the two subthemes was changed future life prospects because of the measures.

#### 3.3.1 Worries about future education

Numerous youths worried about their future education. They were afraid that they would not get into the education programme they dreamed of, because their learning outcome and grades could decline as a result of homeschooling. One youth wrote: ‘That it becomes more difficult to get good grades due to everything that happens, and therefore I may not get into the study I want later.’

#### 3.3.2 Worries about economic safety in the future

Many participants were worried about the economic consequences of the pandemic for their future. Some pointed out that it could be difficult to get the job they dreamed of, but far more respondents worried about negative economic consequences for their close family. This was expressed through worry or fear that family members, mainly parents, could lose their job if the lockdown lasted a long time. Still, what raised most concern among the youth was the national and global economic footprint of the pandemic. Some voiced concerns about destabilization of national economics that would influence the youth's possibility to have a safe future. Worries about national and international safety hazards affecting the global economy, and ultimately resulting in less democracy, conflict and war, were also expressed.

One youth said: ‘I do not think the world will be the same when the pandemic is over. The world economy will be worse, and the opportunities may be fewer. I also believe that this will affect everyone in the sense that we may be more afraid of infection in the future. That the economy will be destroyed and that the adults of the future will have a lot of work to rebuild it’.

## 4 DISCUSSION

This study explores worries about the future among youth during the initial COVID‐19 lockdown in Norway. Our findings build on the responses to an open‐ended survey question from a large number of youths giving unique insight, breadth and credibility to their experienced worries. The worries covered three main themes: (i) ‘That my mom dies, then I am left all alone’: worries related to COVID‐19 virus infection; (ii) ‘To me, this is lost youth’: worries about the consequences of measures for the present life and near future; and (iii) ‘I will face a very difficult life in the future’: worries about the consequences of measures for the outlook on life.

The results suggest that few worried about the possibility of themselves getting infected by COVID‐19, but rather that parents or grandparents in medical risk groups would become seriously ill and die. This finding aligns with recent research on worries among youth in Norway (Dyregrov et al., 2020), and in Italy (Commodari & La Rosa, 2020), and provides a deeper understanding of the quantitative results from COVID‐19 Young (Lehmann et al., 2021), where a total of 53% worried that someone in their family would get infected. The results presented in this paper suggest that such worries were founded in basic fear of separation and bereavement of primary attachment figures, revealing a profound anxiety of being left alone, unprotected. Many of the youths describing these worries had parents with somatic ill‐health. In that perspective, one might view the pandemic as accentuating fears that children of somatically fragile parents often carry with them. There were no comments in our material about worries that parents working in health‐care or other frontline positions during the pandemics could be infected as found by other researchers (ECDC, 2020).

Most of the comments concerned the consequences of measures implemented to control the spread of the virus. Even if the data were collected during the seventh to ninth week of the lockdown, the comments gave the impression that youth suspected that this was not a short‐term medical crisis; they worried about long‐term consequences. Many saw this period as an irrevocable loss of adolescence and worried about losing significant life events and rites of passage to adult life. But also, that closed schools implied a loss of social meeting points and worries about their mental health. The findings suggest that the social distancing as a measure radically reducing adolescents' opportunities to keep physical social contact with peers outside their household. Social connectedness and social identity have more prominence in youth. Adolescents are particularly vulnerable to isolation from peers, as they are in the developmental stage where the peer group is of particular importance for identity development (Blakemore & Mills, 2014; Brown et al., 1986; Orben et al., 2020).

Our results underscore the potential serious mental health impacts for youth undergoing COVID‐19 restrictions. Feelings of loneliness due to social distancing, and school closure, were highlighted by a substantial proportion of the participants. This is in line with findings from a rapid review stating a consistent relationship between loneliness and poor mental health during and after enforced isolation ends among children and adolescents (Loades et al., 2020). Those with mental health problems prior to the pandemic and those in vulnerable family situations expressed most concerns about the lack of health and social services during lockdown, and the psychological tolls of school closure. Thus, it seems that the young people's perceptions were affected by pre‐existing vulnerabilities such as living with a disability or pre‐existing mental health problems. Our findings align with other studies showing that the impacts of COVID‐19 are unevenly distributed among young people (Rogers et al., 2021). The function of the school is described not just as an arena for academic growth. The importance of the school context and relations with teachers, as well as fellow students, for their recovery or as part of a therapeutic regime is highlighted through our findings. Further, there were alarming descriptions of increased family tension in some of our material. Taken together, our findings are in line with increasing concerns that the closing of schools and limited social contact outside of close family poses a significant threat for adolescents already facing a range of different risk factors, such as caring responsibilities, mental health challenges, learning difficulties or belonging to vulnerable households (Masonbrink & Hurley, 2020; Orben et al., 2020; Rogers et al., 2021). Moreover, our material shows that also youth with no pre‐existent mental health issues worried about developing such problems.

Youth were concerned that they would not get into the education program they aspired to, because their learning outcome and grades could decline because of homeschooling. They were also concerned that it would be negative economic consequences of the pandemic for their own future. School‐ and academic failure are part of worries in everyday life for adolescents (Gilman & Huebner, 2006; Matos et al., 2016). Based on the qualitative results and supported by the quantitative data (Lehmann et al., 2021), this worry was even more evident under the COVID‐19 lockdown. Coping strategies used by youth are often associated with close contact with family, peers (social support) and leisure time (distraction) (Matos et al., 2016). According to the stress‐buffer theory, friends play a central role as a relatively stable source for social support, which can protect people from the negative effects of stressful events primarily by influencing appraisal and coping (Cohen & Wills, 1985). When strict social measures for infection control are introduced, such as physically closed schools, social support and distraction as coping strategies become less available. The results from this study show that youth themselves already in the first weeks of the pandemic saw this as a threat to both present life and the more distant outlook on life. According to Power et al. (2020), young people may also find it more difficult to cope with the current crisis as their coping skills are not equivalent to that of an adult.

### 4.1 Limitations

Readers should be mindful that youth responding to the survey tended to come from families with higher SES and thus may not be completely representative for their age group in Norway.

The use of an open‐end question intended to bring out the voices of young people themselves, to understand and broaden the quantitative findings on young peoples' worries (Lehmann et al., 2021). However, one limitation is that respondents already had replied to several survey questions on possible worries during the pandemic. It might be that the method itself (open‐end questions in a questionnaire) significantly shaped responses such as replication to the a priori response alternatives. However, Singer and Couper (2017) suggest that this method may empower and motivate respondents, because it gives them an opportunity to express their views on the topics addressed in the survey in their own words. Allemang et al. (2021) are advocating that open‐ended questions are suitable to capture youth perspective during the pandemic, because it captures a broad spectre of responses since a survey could be administered to a large sample of the target population.

However, because the expressions were not collected from dialogue with the youth, we missed the opportunity for further nuances that could have clarified and elaborated their written answers (Kvale, 1989), such as statements that would allow youth to express other (including neutral or positive) aspects. We ensured that there were no character limitations to the question. Still, what we found was text based on short sentences and keywords rather than long stories. Also, an avenue for future research could be to follow‐up youth responding that they were not worried. This could give an interesting insight into supportive factors and resilience beyond the findings from this study. With more people being vaccinated, and a gradual reopening of the society, further research is also needed to illuminate if or how this impacts on the worries of young people.

Finally, our study focuses on worries as an aversive emotional experience among youth, but for some, this period may also receive some benefits. It has been argued that worries have an upside and that it leads to motivational benefits and work as an emotional buffer (Sweeny & Dooley, 2017). This should be explored further within the framework of resilience theory in the future.

### 4.2 Concluding remarks and implications for practice

International research claims that it is expected that the global crisis caused by the COVID‐19 pandemic, including the disease and prolonged social distancing, will have major impacts on young peoples' life and future (ECDC, 2020; Loades et al., 2020). Scholars have advocated that young people may be more affected by the negative psychosocial consequences of ‘lockdown’ and social distancing than adults, as social identity and connectedness are prominent in this phase of life. The youths' worries about the longstanding consequences of being deprived of social and educational opportunities, and potential mental health impacts on short and long terms are consistent with this view. The results from COVID‐19 Young conducted in a Norwegian context complement and align with research from other countries (ECDC, 2020; Masonbrink & Hurley, 2020; Nøkleby et al., 2021; Orben et al., 2020; Rogers et al., 2021) and highlight the urgency to rethink and plan delivery of educational, social and health services and possibilities to meet the needs of this cohort. Specifically, social and mental health services for youth should offer early interventions and be prepared for an escalation in mental health problems in the imminent future. The results from this study concur with previous findings that certain groups of young people seem particularly vulnerable and should be given particular attention.

Beyond the urgency for dedicated health services, our findings underscore that the threshold for closing schools and replace it with distance learning should be kept high. We follow Shukla et al. (2021) advocating the need for investment of resources into a safe opening of schools. Remote teaching and digital technology such as the internet, personal computers, tablets and mobile phones may be suitable for emulating classroom‐like interactions (Bubb & Jones, 2020). Nonetheless, schools are a social meeting point for youth. For some, it provides crucial opportunities to interact with adults outside of their household and to be exposed to social interaction otherwise lost to them. By closing schools, important identity development, coping strategies and learning outcomes are put to risk. Many of the youth participating in this study saw the potential that the measures could have a subsequential chain of negative impact on their possibility to fulfil their educational dreams and economic safety in the future.

As the pandemic and the ongoing measures to prevent the spreading of the disease has continued, the degree and nature of youth worries may look different with time. Ongoing research to understand the impact at different stages of the pandemic is important to inform the society. International multidisciplinary research highlights that psychological resilience is crucial to cope with the COVID‐19 pandemic implications (Masten & Motti‐Stefanidi, 2020). This aspect should be followed into future research, where increased focus on the processes that foster a positive cascade of resilience for youth in a pandemic is needed (Masten & Motti‐Stefanidi, 2020).

According to the United Nations Children’s Fund UK (1989), the voice of young people should be heard and valued. During and after COVID‐19, they should be looked upon as an essential resource in the evaluation of the current suppression strategies and for the planning of future plans to repress pandemics. Based on the dissemination of young peoples experience, the government, policymakers, health and educational professionals could be better prepared to meet the future global crises but also everyday life during the COVID‐19 pandemic. To sum up, one youth said: ‘How will the future be after COVID‐19? We are unlikely to return to the same world, and I do not want it to destroy my future’.

## CONFLICT OF INTEREST

The authors declare that they have no conflict of interest.

## Data Availability

The data that support the findings of this study are available on request from the corresponding author. The data are not publicly available due to privacy or ethical restrictions.
